# Vaccination coverage in children up to 2 years old born in 2017 and 2018 in the municipalities of São Paulo and Campinas, Brazil: comparison of the results of a national survey and the National Immunization Program Information System

**DOI:** 10.1590/S2237-96222024v33e2023539.especial2.en

**Published:** 2025-01-10

**Authors:** Manoela Alves Algodres, Ana Paula França, José Cássio de Moraes, ICV 2020 Group, Adriana Ilha da Silva, Alberto Novaes Ramos, Ana Paula França, Andrea de Nazaré Marvão Oliveira, Antonio Fernando Boing, Carla Magda Allan Santos Domingues, Consuelo Silva de Oliveira, Ethel Leonor Noia Maciel, Ione Aquemi Guibu, Isabelle Ribeiro Barbosa Mirabal, Jaqueline Caracas Barbosa, Jaqueline Costa Lima, José Cássio de Moraes, Karin Regina Luhm, Karlla Antonieta Amorim Caetano, Luisa Helena de Oliveira Lima, Maria Bernadete de Cerqueira Antunes, Maria da Gloria Teixeira, Maria Denise de Castro Teixeira, Maria Fernanda de Sousa Oliveira Borges, Rejane Christine de Sousa Queiroz, Ricardo Queiroz Gurgel, Rita Barradas Barata, Roberta Nogueira Calandrini de Azevedo, Sandra Maria do Valle Leone de Oliveira, Sheila Araújo Teles, Silvana Granado Nogueira da Gama, Sotero Serrate Mengue, Taynãna César Simões, Valdir Nascimento, Wildo Navegantes de Araújo

**Affiliations:** 1 Faculdade de Ciências Médicas da Santa Casa de São Paulo, Departamento de Saúde Coletiva, São Paulo, SP, Brasil; Universidade Federal do Espírito Santo, Vitória, ES, Brazil; Universidade Federal do Ceará, Departamento de Saúde Comunitária, Fortaleza, CE, Brazil; Faculdade Ciências Médicas Santa Casa de São Paulo, São Paulo, SP, Brazil; Secretaria de Estado da Saúde do Amapá, Macapá, AP, Brazil; Universidade Federal de Santa Catarina, SC, Brazil; Organização Pan-Americana da Saúde, Brasília, DF, Brazil; Instituto Evandro Chagas, Belém, PA, Brazil; Faculdade de Ciências Médicas Santa Casa de São Paulo, Departamento de Saúde Coletiva, São Paulo, SP, Brazil; Universidade Federal de Mato Grosso, Cuiabá, MT, Brazil; Universidade Federal do Paraná, Curitiba, PR, Brazil; Universidade Federal de Goiás, Goiânia, GO, Brazil; Universidade Federal do Piauí, Teresina, PI, Brazil; Universidade de Pernambuco, Faculdade de Ciências Médicas, Recife, PE, Brazil; Instituto de Saúde Coletiva, Universidade Federal da Bahia, Salvador, BA, Brazil; Secretaria de Estado da Saúde de Alagoas, Maceió, AL, Brazil; Universidade Federal do Acre, Rio Branco, AC, Brazil; Universidade Federal do Maranhão, Departamento de Saúde Pública, São Luís, MA, Brazil; Universidade Federal de Sergipe, Aracaju, SE, Brazil; Secretaria Municipal de Saúde, Boa Vista, RR, Brazil; Fundação Oswaldo Cruz, Mato Grosso do Sul, Campo Grande, MS, Brazil; Fundação Oswaldo Cruz, Escola Nacional de Saúde Pública Sergio Arouca, Rio de Janeiro, RJ, Brazil; Universidade Federal do Rio Grande do Sul, Porto Alegre, RS, Brazil; Fundação Oswaldo Cruz, Instituto de Pesquisa René Rachou, Belo Horizonte, MG, Brazil; Secretaria de Desenvolvimento Ambiental de Rondônia, Porto Velho, RO, Brazil; Universidade de Brasília, Brasília, DF, Brazil

**Keywords:** Cobertura de Vacunación, Salud Infantil, Encuestas Epidemiológicas, Programas de Imunización, Vaccination Coverage, Child Health, Health Surveys, Immunization Programs

## Abstract

**Objective:**

To estimate and compare vaccination coverage among children born in 2017-2018 in São Paulo and Campinas, according to the Vaccination Coverage Survey (ICV 2020) and the National Immunization Program Information System (SI-PNI).

**Methods:**

ICV 2020 analyzed vaccination card records. Coverage was calculated and compared to doses recorded on the SI-PNI, divided by the target population.

**Results:**

In São Paulo, according to ICV, in 2017 only BCG (91.7%; 95%CI 87.0;94.7) and rotavirus first dose (90.6%; 95%CI 86.5;93.5) achieved the goals; in 2018, BCG (93.4%; 95%CI 89.5;95.8), rotavirus first dose (90.5%; 95%CI 85.3;94.0), pneumococcal first dose (95.3%; 95%CI 91.7;97.4), meningococcal C first dose (95.1%; 95%CI 91.5;97.2) and pneumococcal second dose (95.0%; 95%CI 91.4;95.0). In Campinas, only BCG achieved the target in 2017 (93.0%; 95%CI 88.8;95.7) and none in 2018. According to the SI-PNI, no vaccine achieved the target in either city.

**Conclusion:**

Vaccination coverage was lower than expected and more precise estimates are necessary for adequate monitoring of childhood vaccination status.

## Study contributions

**Main results:**

Vaccination coverage fell short of targets in both data sources (vaccination survey and information system), which puts control of vaccine-preventable diseases in both cities at risk.

**Implications for services:**

Highlighting the importance of accurate monitoring for control of vaccine-preventable diseases and enabling strategies to maintain high vaccination coverage.

**Perspectives:**

It is essential to provide more incentives for implementation and/or addressing information system shortcomings in order to achieve adequate monitoring of vaccination status.

## RESUMEN

**Objetivo:**

Estimar y comparar la cobertura de vacunación en nacidos entre 2017-2018 en los municipios de São Paulo y Campinas, según la Encuesta de Cobertura de Vacunación (ICV 2020) y el Sistema de Información del Programa Nacional de Inmunizaciones (SI-PNI).

**Métodos:**

ICV 2020 analizó registros históricos de los cuadernos de vacunación. Se calculó la cobertura y se comparó con las dosis registradas en SI-PNI, divididas por la población-objetivo.

**Resultados:**

En São Paulo, según ICV, en 2017 sólo BCG (91,7%; IC95% 87,0;94,7) y rotavirus primera dosis (90,6%; IC95% 86,5;93,5) alcanzaron las metas; en 2018, BCG (93,4%; IC95% 89,5;95,8), primera dosis de rotavirus (90,5%; IC95% 85,3;94,0), neumococo (95,3%; IC95% 91,7;97,4) y meningococo C (95,1%; IC95% 91,5;97,2); y neumocócica segunda dosis (95,0%; IC95% 91,4;95,0). En Campinas, sólo BCG alcanzó la meta en 2017 (93,0%; IC95% 88,8;95,7) y ninguna en 2018. Según SI-PNI, ninguna alcanzó la meta en ambas ciudades.

**Conclusión:**

La cobertura de vacunación fue menor a lo esperado; se necesitan estimaciones más precisas para un adecuado seguimiento de vacunación infantil.

## INTRODUCTION

Having knowledge of child vaccination coverage provides important information, such as evaluating the effectiveness and efficiency of the National Immunization Program (*Programa Nacional de Imunizações* - PNI). This program was established in 1973 and institutionalized in 1975, with the aim of coordinating systematic vaccination actions at the national level, thus intensifying immunization activities in Brazil.^1,2^ Incorporation of new vaccines into the routine schedule has increased the complexity of the PNI in last two decades and has brought new challenges, such as achieving and maintaining high vaccination coverage.^3,4^

Since the creation of the PNI, important progress has been made with its information systems, moving on from initial manual data through to computerization. From 1980 onwards, data began to be consolidated on Excel spreadsheets regarding doses administered as per the national schedule which, at the time, included vaccines against diphtheria, tetanus and pertussis (DTP), measles, oral poliovirus and Bacillus Calmette-Guérin (BCG). The data was limited and compiled by the country’s federative units. The process of computerizing vaccination data in Brazil began in 1994.^5^

With the support of the Brazilian National Health System Information Technology Department, the Immunization Program Evaluation Information System (*Sistema de Informação de Avaliação do Programa de Imunizações* - SIAPI) was developed, containing vaccination records according to doses administered, aggregated by type of immunobiological product, target group, time and place, and was the only official vaccination information system in Brazil until 2012. That year, the SIAPI was enhanced and put on a Web platform (SIAPIWeb) and remained in force for routine vaccination records until December 2020, in some municipalities, while others implemented nominal vaccination records.^5^ SIAPIWeb was used until the National Immunization Program Information System (*Sistema de Informação do Programa Nacional de Imunizações* - SI-PNI) was fully implemented. An innovation brought by this system was individualized (nominal) records with the aim of correcting limitations related to the aggregated data system, in order to improve the accuracy of vaccination indicators. Until 2019, 85% of the 38,000 vaccination rooms used the SI-PNI Vaccination Record module.^5^

In recognition of the evolution and process of improving the system, it is worth highlighting that SI-PNI data enable estimation of vaccination coverage based on the number of doses administered, but do not reveal individual vaccination status or the percentage of children with a full schedule. Other limitations, such as errors in estimating the size of the target population in intercensal years, can underestimate or overestimate the denominator, which generates distorted vaccination coverage data.^6^

Data from household surveys produce more accurate estimates of coverage and make it possible to estimate the number of children with a full schedule. These data also contribute to understanding the socioeconomic determinants associated with heterogeneous distribution of childhood vaccination coverage and to estimating the proportion of susceptible children and factors related to equity of access to the PNI.^6^

Currently, the PNI faces the challenge of recovering the achievement of vaccination coverage targets and, to this end, it is essential that information systems provide more accurate data. In Brazil, the decline in child vaccination schedule coverage began in 2012, becoming more pronounced from 2016 onwards. In 2020, the COVID-19 pandemic worsened this scenario.^7^

The most recent national Vaccination Coverage Survey (*Inquérito de Cobertura Vacinal* - ICV 2020) was conducted in the capital cities of Brazil’s 26 states, Federal District and also in 12 interior region municipalities. It included 37,801 children born between 2017 and 2018 who lived in urban areas. The ICV 2020 found that only 60.1% had received all the vaccines that make up the vaccination schedule to be administered to children by the time they are 24 months old.^8^

Studies that compared official administrative data with data from vaccination coverage surveys have indicated important differences in vaccination coverage, which indicate possible flaws in recording administered doses and/or estimating the target population.^9^

The objective of this study was to estimate and compare vaccination coverage among children up to 2 years old born between 2017 and 2018 in the municipalities of São Paulo and Campinas, Brazil, according to ICV 2020 data and SI-PNI data. 

## METHODS

This study used two data sources to calculate and compare PNI vaccination schedule coverage among children up to 24 months old, namely ICV 2020 and SI-PNI, for children born in 2017 and 2018, in the municipalities of Campinas and São Paulo, located in the state of São Paulo, Brazil. Both cities are large, with populations estimated at 1,139,047 and 11,451,999 inhabitants, respectively, according to the 2022 Demographic Census conducted by the Brazilian Institute of Geography and Statistics (*Instituto Brasileiro de Geografia e Estatística*).^10^

### ICV 2020

ICV 2020 is a population-based survey based on a retrospective cohort of child vaccination from birth to 24 months old. Sampling began with stratification, in each estimation domain, based on 2010 Demographic Census tract information, which generated four socioeconomic strata for the urban sectors of the municipalities (A, B, C and D, whereby A accounted for the best socioeconomic conditions, while D accounted for the poorest). The stratification cutoff points for each municipality were different, and considered the nominal income of the head of the family, the percentage of heads of family with income greater than 20 minimum wages and the percentage of heads of family who were literate.

The list of newborns, obtained via the Ministry of Health, was georeferenced according to the census tracts where their mothers lived. The sample size calculation was based on 70% vaccination coverage, design effect of 1.4 and a 95% confidence level, resulting in 452 newborns in each stratum, totaling a predicted sample size of 1,808 children in each municipality. More in-depth methodological details have been described in a previous publication.^8^

Data collection took place by means of home visits, from June 2020 to May 2022, with trained interviewers who recorded answers to a structured questionnaire and saved photographs of the children’s vaccination cards on a tablet. 

Coverage of all vaccines to be administered by 24 months of age according to the official PNI schedule was calculated: BCG; hepatitis B (HepB); DTP + *hemophilus influenza* B + hepatitis B (DTP-Hib-HepB)first + second + third dose; inactivated poliovirus 1, 2 and 3 (IPV: first + second + third dose); rotavirus (RV1: first + second dose); meningococcal serogroup C (MENC: first + second dose + booster); pneumococcal conjugate (PCV10: first + second doses + booster); yellow fever (YF), measles, mumps and rubella (MMR: first + second doses); hepatitis A (HepA: first dose); varicella (VAR: single dose); poliovirus 1 and 3 (attenuated) (bOPV: booster); and diphtheria, tetanus and pertussis (DTP: booster). 

Considering that different vaccine compositions are used to protect against the same diseases, a variable was combined for each dose of vaccine that forms part of the PNI schedule.^11^ An example is the calculation of the coverage of pneumococcal conjugate vaccine (PCV10, administered in public services): PCV10 3^rd^ doses were added together, in order to account for vaccines administered by both public and private services.

Vaccination coverage according to administered doses and respective confidence intervals was calculated using the Stata®, version 17, survey analysis module. 

The ICV 2020 was approved by the Research Ethics Committee of the *Instituto de Saúde Coletiva da Universidade Federal da Bahia*, as per Opinion No. 3.366.818, on June 4, 2019, and Certificate of Submission for Ethical Appraisal (*Certificado de Apresentação de Apreciação* Ética - CAAE) No. 4306919.5.0000.5030; and by the Research Ethics Committee of the *Irmandade da Santa Casa de Misericórdia de São Paulo*, as per Opinion No. 4.380.019, on November 4, 2020, and CAAE No. 39412020.0.0000.5479.

### SI-PNI

The number of administered doses of each immunization product comprising the current vaccination schedule was obtained in October 2023, from the SI-PNI website,^12^ for the years 2017, 2018 and 2019, in each age group (<1 year and 1 year) for live births in 2017 and 2018.

Vaccination coverage was calculated by dividing the number of doses of a given administered vaccine by the estimate of its target population, multiplied by 100. Data from the Live Birth Information System (*Sistema de Informações sobre Nascidos Vivos*)^13^ indicate that there were 189,740 and 185,016 live births in São Paulo and 21,651 and 21,339 live births in Campinas, in 2017 and 2018, respectively.

Different vaccine combinations are recorded on the SI-PNI, as is the case of MMR and measles, mumps, rubella and varicella (MMRV). In order to calculate the total number of doses administered, it was necessary to add together the doses of the different immunization products used to protect against the same diseases, in accordance with guidelines issued by the Health Ministry Health Surveillance Secretariat.^14^

As our study used public domain anonymized and aggregated ICV 2020 data with unrestricted access, presented in a consolidated manner by municipality, year and age group, Research Ethics Committee appraisal was not necessary.

### PNI performance indicators

The vaccination coverage and dropout rate indicators were used to compare ICV and SI-PNI data.

### Vaccination coverage 

For both data sources, vaccination coverage that reached values equal to or greater than 90% for BCG and RV1 and 95% for the other vaccines was considered adequate.^5^

### Dropout rates 

Dropout rates were calculated by dividing the number of children who started but did not complete the vaccine schedule by the number of first doses administered, multiplied by 100. The rates were considered to be: high, if greater than 10%; medium, if between 5% and 10%; and low, if less than 5%.^14^

## RESULTS

Data collection took place from July 2021 to February 2022, in the municipality of São Paulo, and between October 2021 and April 2022, in Campinas. The ICV sample was made up of 797 and 742 children born in the city of São Paulo (85.1% of the calculated sample size) and 912 and 862 born in Campinas (98.1% of the calculated sample size) in 2017 and 2018, respectively.

### Municipality of São Paulo, children born in 2017

**Figure 1:**
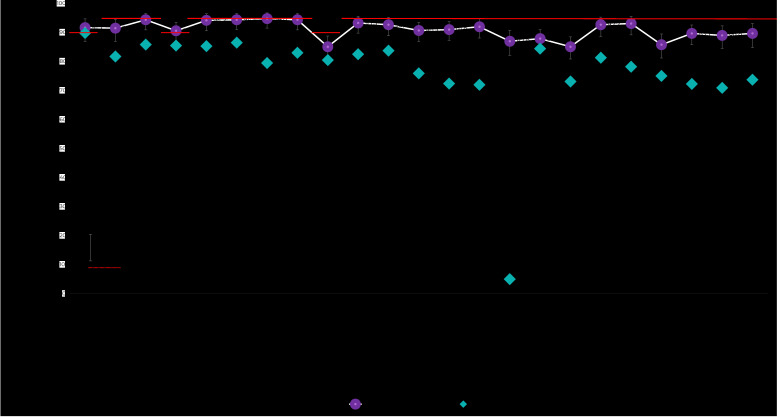
Coverage of vaccines recommended by the basic vaccination schedule, according to data from the Vaccination Coverage Survey (ICV 2020) and from the National Immunization Program Information System (SI-PNI), differences between the sources (ICV 2020 *versus* SI-PNI) and regarding coverage targets (PNI), among children born in 2017 in the municipality of São Paulo

**Figure 2:**
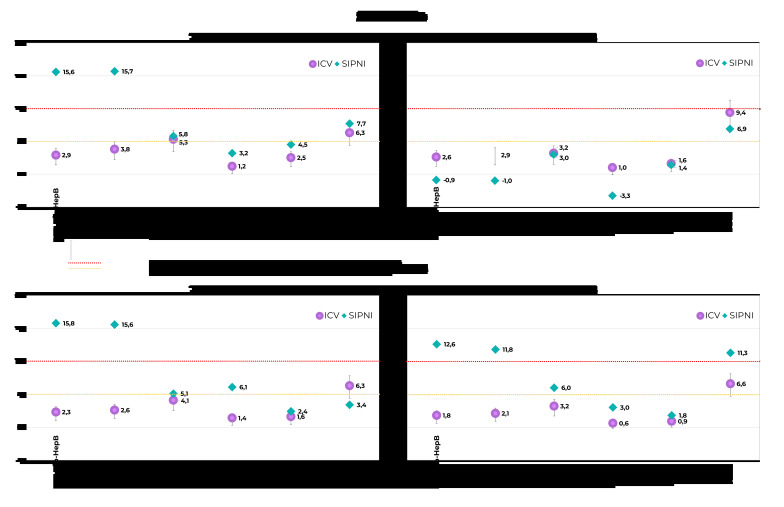
Dropout rates for measles, mumps and rubella (MMR), meningococcal serogroup C (MENC), pneumococcal conjugate (PCV10), DTP-Hib-HepB, rotavirus (RV1) and inactivated poliovirus 1, 2 and 3 (IPV) vaccine schedules, according to data from the Vaccination Coverage Survey (ICV 2020) and from the National Immunization Program Information System (SI-PNI) among children born in 2017 and 2018 in the municipalities of São Paulo and Campinas

According to the ICV, only BCG vaccine coverage (91.7%; 95%CI 87.0;94.7%) and rotavirus vaccine coverage (90.6%; 95%CI 86.5;93.5%) achieved the PNI targets. On average, coverage was 3.4 percentage points (p.p.) below recommended levels, ranging from +1.7 p.p. for BCG to -9.9 p.p. for the MENC booster (Figure 1). Dropout rates were medium for the MMR and RV1 vaccine schedules, and no schedule had a high dropout rate (Figure 2).

According to the SI-PNI, only BCG vaccine coverage came close to the target (89.8%). The target was not met by any of the vaccines. Variation was between -0.2 p.p. for BCG and -24.1 p.p. for bOPV; while average variation was -17.9 p.p. (Figure 1). Dropout rates were high for the DTP-Hib-HepB and IPV schedules and medium for the MMR and RV1 schedules (Figure 2).

For all vaccines, ICV coverage was higher than coverage calculated using SI-PNI data. When examining the 95% confidence intervals, the only vaccine coverage that was not different between the two sources referred to BCG, RV1 first dose and the PCV10 booster. Average coverage difference was +14.4 for the ICV; whereby the lowest difference was found for BCG (+1.9 p.p. for the ICV) and the highest difference was found for YF (+82.0 p.p. for the ICV) (Figure 1).

### Municipality of São Paulo, children born in 2018

With regard to the ICV, only the coverage of BCG, the first dose of RV1, the first dose of PCV10, the first dose of MENC and the second dose of PCV10 achieved the PNI targets: 93.4% (95%CI 89.5;95.8%), 90.5% (95%CI 85.3;94.0%), 95.3% (95%CI 91.7;97.4%), 95.1% (95%CI 91.5;97.2%) and 95.0% (95%CI 91.4;95.0%), respectively. On average, coverage was 2.8 p.p. below the recommended targets. Dropout rates were high for the MMR vaccine and medium for PCV10 (Figure 2).

**Figure 3:**
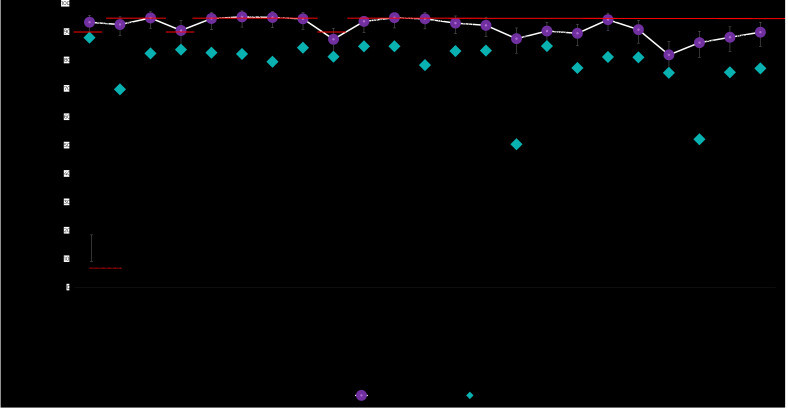
Coverage of vaccines recommended by the basic vaccination schedule, according to data from the Vaccination Coverage Survey (ICV 2020) and from the National Immunization Program Information System (SI-PNI), differences between the sources (ICV 2020 *versus* SI-PNI) and regarding coverage targets (PNI), among children born in 2018 in the municipality of São Paulo

According to the SI-PNI, none of the vaccines achieved the target, as was also found for those born in 2017, as per the same data source. On average, coverage rates were 15.9 p.p. below recommended rates (Figure 3). Dropout rates were medium for the MMR vaccine schedule and negative for PCV10 and DTP-Hib-HepB, with more records of the last than the first dose of the schedule, in the same age group (Figure 2).

Coverage rates found by the ICV were higher than those recorded on the SI-PNI for children born in 2018 (p < 0.05), for all vaccines. The average difference between the two sources was +13.1 p.p., for the ICV. The lowest difference was found for the PCV10 booster (+5.3 p.p. for the ICV), while the highest difference was found for YF (+37.2 p.p. for the ICV) (Figure 3). 

The ICV showed a slight improvement (+0.7 p.p.) when compared to average vaccination coverage among those born in 2017 and 2018 in the municipality of São Paulo. The highest differences were found for the MENC vaccine booster (+4.4 p.p.) and the second dose of the MMR vaccine (-3.9 p.p.); considering the 95% CIs, there was no statistical difference between coverage in the two periods. 

According to the SI-PNI, between 2017 and 2018 there was a +2.0 p.p. increase in average coverage, with large variations: from -20.1 p.p. for DTP to +45.4 p.p. for YF (Figures 1 and 3).

### Municipality of Campinas, children born in 2017

**Figure 4:**
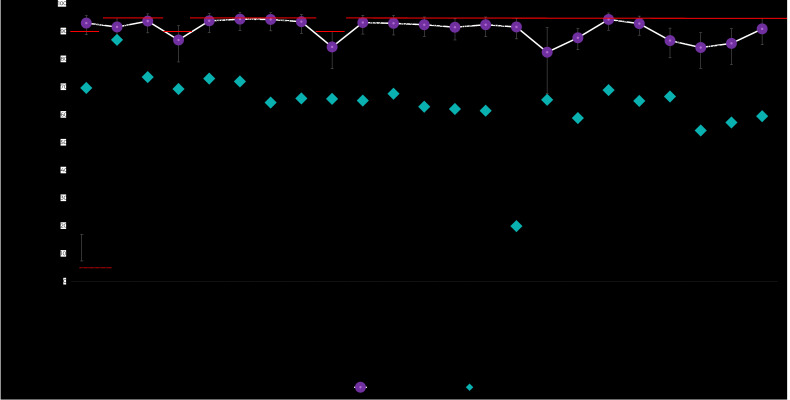
Coverage of vaccines recommended by the basic vaccination schedule, according to data from the Vaccination Coverage Survey (ICV 2020) and from the National Immunization Program Information System (SI-PNI), differences between the sources (ICV 2020 *versus* SI-PNI) and regarding coverage targets (PNI), among children born in 2017 in Campinas

With regard to the ICV, only BCG vaccine coverage (93.0%; 95%CI 88.8;95.7%) achieved the target. On average, coverage was 3.7 p.p. below target, ranging from +3.0 p.p. for BCG to -12.5 p.p. for the first PCV10 booster (Figure 4). Dropout rates were medium for the MMR vaccine schedule and low for the other schedules (Figure 2).

**Figure 5:**
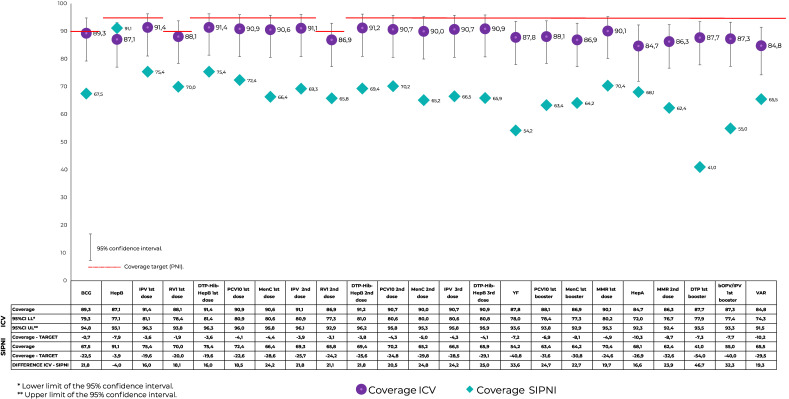
Coverage of vaccines recommended by the basic vaccination schedule, according to data from the Vaccination Coverage Survey (ICV 2020) and from the National Immunization Program Information System (SI-PNI), differences between the sources (ICV 2020 *versus* SI-PNI) and regarding coverage targets (PNI), among children born in 2018 in Campinas

According to the SI-PNI, no vaccination coverage achieved the target, with an average difference of -30.2 p.p., varying from -8.0 p.p. for HepB to -75.1 p.p. for YF (Figure 3). Dropout rates were high for the DTP-Hib-HepB and IPV schedules and medium for the PCV10 and RV1 schedules (Figure 5).

The difference between the coverage found by the two sources was also large, on average +26.5 p.p. for the ICV. The lowest difference was found for the HepB vaccine (+4.6 p.p. for the ICV), while the highest difference was found for YF (+71.7 p.p. for the ICV), followed by varicella (+31.4 p.p. for the ICV) (Figure 4). Considering the 95% CIs, only HepB coverage showed no statistical difference between the two sources.

### Municipality of Campinas, children born in 2018

According to the ICV, none of the vaccine targets were achieved for vaccination by 24 months old. The smallest difference in relation to the targets was found for BCG (-0.7 p.p.), while the largest differences were found for VAR (-10.2 p.p.) and HepA (-10.3 p.p.) (Figure 4). Dropout rates were low for all schedules (Figure 2).

According to the SI-PNI, the average difference in target coverage was -27.6 p.p., being lower for the HepB vaccine (-3.9 p.p.) and higher for bOPV (-40.0 p.p.), YF (-40.8 p.p.) and the DTP booster (-54.0 p.p.) (Figure 5). Dropout rates were high for the MMR and DTP-Hib-HepB vaccine schedules and medium for RV1 vaccine (Figure 5).

As found for children born in 2017, the ICV found coverage higher than that recorded on the SI-PNI for children born in 2018 for almost all vaccines, with the exception of the HepB vaccine (+4 p.p. for the SI-PNI). On average, ICV found coverage 22.1 p.p. higher than coverage recorded by the SI-PNI, with greater differences for bOPV (+32.3 p.p. for the ICV), yellow fever (+33.6 p.p. for the ICV) and DTP (+46.7 p.p. for the ICV) (Figure 4). 

Contrary to what was found in São Paulo, most coverage rates were lower among children born in 2018 in Campinas, according to ICV data (Figures 4 and 5). The average difference was -1.8 p.p., and the biggest differences were for the PCV10 booster (+5.6 p.p.) and for VAR (-6.1 p.p.); considering the 95% CIs, there was no statistical difference between vaccine coverage in the two years. According to the SI-PNI, coverage among children born in 2018 was better (+2.6 p.p.), with the biggest differences found for the YF vaccine (+34.3 p.p.) and the DTP vaccine (-13.3 p.p.)). 

## DISCUSSION

In the city of São Paulo, according to the ICV, only two vaccines met the targets in 2017 and only five in 2018, including BCG. In Campinas, only BCG met the target in 2017 and no vaccines achieved the targets in 2018. When using SI-PNI data, none of the vaccines achieved the coverage target among children up to 2 years old born in 2017 and 2018 in either of the cities. 

Time series of vaccination coverage among children in Brazil based on SI-PNI data have shown that from 2000-2015, all targets recommended by the Ministry of Health were achieved,^15^ while from 2016-2018 only BCG met the target.^16^ Between 2015 and 2017, the biggest falls in vaccination were recorded for poliovirus (-21.2%), hepatitis A (-21.0%), meningococcal serogroup C (-19.5%), rotavirus (-20.2%), DTP-Hib-HepB (-19.8) and hepatitis B (-17.6%) vaccines.^17^

It is important to emphasize that this phenomenon is not localized. A comprehensive assessment of global coverage patterns for 11 vaccines in 204 countries highlighted remarkable progress between 1980 and 2010, the period in which the greatest proportion of children were protected from vaccine-preventable diseases in the history of vaccination against diseases. However, between 2010 and 2019, a period in which the development of new vaccines was largely successful, the gain in vaccination coverage was minimal. In some places, especially in Latin America and the Caribbean, vaccination coverage has fallen.^18^

These drops can be attributed to several factors, driven by anti-vaccine movements^19^ with the spread of fake news that places doubt on effectiveness and attributes serious adverse reactions to vaccines.^20^ There is also a lack of concern regarding diseases that are considered to have been eliminated or eradicated, as many believe that vaccination against them is no longer necessary, assuming that these diseases would not pose a threat to children’s health.^21^

It needs to be taken into consideration that shortage of some products, operational challenges in properly performing vaccination, including accurate recording of data, and difficulties in accessing health services are elements that may explain low vaccination coverage. Understanding these factors is essential for planning new approaches to recovering the high vaccination coverage rates previously achieved.^17,22^

Comparing the two data sources, coverage of all vaccines required by the infant schedule for children up to 24 months old was lower when calculated using SI-PNI data for live births in 2017 and 2018, in both municipalities, except for hepatitis B vaccine coverage in Campinas in 2018. These results highlight difficulties in the computerized recording system, which reduce the accuracy of reported childhood vaccination coverage. 

Despite all the progress achieved, the various areas of the PNI face major challenges, due to the complexity of its entire trajectory. Improved immunization data in Brazil has developed with the use of the SI-PNI, but considerable problems are still being identified, which may affect the quality of the data recorded.^16^ The SI-PNI is in an advanced phase of implementation in Brazil, however, it faces technical, bureaucratic and operational challenges, related to training health professionals, mastering technology, maintaining the system and guaranteeing the confidentiality of information.^23^

Even with incentives for implementing the system and support for training provided by the Ministry of Health, there has been no massive adoption of using the SI-PNI by municipal health services. There is still resistance to recording data individually, as the previous system of recording aggregate doses was faster. The main obstacles reported by state and municipal health departments include difficulty in transmitting data to the national database and slow processing by the Brazilian National Health System Information Technology Department. This happens, in part, due to the SI-PNI being incompatible with the systems used by some municipalities.^15^

The need for internet access by all vaccination rooms, adequate and timely recording of vaccinations when internet access to the system is not available, and preventing duplicated records are among the main difficulties for municipalities to adhere to the system. There are cases of vaccination rooms in the same municipality not sharing information with each other, which can result in someone being registered and vaccinated in more than one health center.^23^

Due to the greater complexity of recording vaccinations via the system, irregular, inadequate and untimely data input can take place, that is, dose administration may not be recorded or may be recorded late. There are also recurring typing errors that compromise information quality. These inconsistencies can result in disparities between local data and consolidated numbers at the national level,^15^ in addition to recording inaccuracies. This impacts immunization indicators, resulting in underestimated coverage.

Emphasis must be given to the importance of training health professionals who process data on the information system in order to obtain more accurate estimates, as this contributes to strengthening the operational capacity of the municipalities. There is a need for permanent training efforts regarding analysis of data input to and exported from the information system.^24^

The results of this study need to be interpreted considering its limitations. With regard to the ICV, data collection took place during the COVID-19 pandemic, which impacted response rates and could affect the accuracy of estimates. It should be noted that the calculation of post-stratification sample weights took into account differences in responses between population groups and minimized them. As the survey only included children from urban areas, this makes it impossible to extrapolate the data, especially in the case of rural populations, small municipalities and minority groups. The main advantage of the survey was its collecting data directly from vaccination cards, by photographing them and the respective data being input by health professionals experienced in the PNI. The data obtained for coverage calculations via the SI-PNI are secondary, subject to possible typing errors, incompleteness or pending input to immunization information systems.

Vaccination coverage among children up to two years old is below the targets established by the PNI. Although well-conducted surveys enable more precise estimates and understanding of the factors that contribute to reduction in vaccination, considerable investment of time and financial resources is required for them to be implemented. It is essential for the information system to be improved in order to provide more accurate estimates, thus enabling adequate monitoring of vaccination status. 
