# Supplementary material for: Vaccination coverage in children up to 2 years old born in 2017 and 2018 in the municipalities of São Paulo and Campinas, Brazil: comparison of the results of a national survey and the National Immunization Program Information System
Source: Epidemiol Serv Saude. 2025 Jan 10;33(spe2):e2023539. doi: 10.1590/S2237-96222024v33e2023539.especial2.en (PMC11734607; doi:10.1590/S2237-96222024v33e2023539.especial2.en)
Supplement: Translation

# Translation: Coberturas vacinais em crianças de até 2 anos de idade nascidas em 2017 e 2018 nos municípios de São Paulo e Campinas: comparação dos resultados de inquérito nacional com os do Sistema de Informação do Programa Nacional de Imunizações

**DOI:** 10.1590/S2237-96222024v33e2023539.especial2.pt

**Authors:** Manoela Alves Algodres, Ana Paula França, José Cássio de Moraes, Grupo ICV 2020, Adriana Ilha da Silva, Alberto Novaes Ramos, Ana Paula França, Andrea de Nazaré Marvão Oliveira, Antonio Fernando Boing, Carla Magda Allan Santos Domingues, Consuelo Silva de Oliveira, Ethel Leonor Noia Maciel, Ione Aquemi Guibu, Isabelle Ribeiro Barbosa Mirabal, Jaqueline Caracas Barbosa, Jaqueline Costa Lima, José Cássio de Moraes, Karin Regina Luhm, Karlla Antonieta Amorim Caetano, Luisa Helena de Oliveira Lima, Maria Bernadete de Cerqueira Antunes, Maria da Gloria Teixeira, Maria Denise de Castro Teixeira, Maria Fernanda de Sousa Oliveira Borges, Rejane Christine de Sousa Queiroz, Ricardo Queiroz Gurgel, Rita Barradas Barata, Roberta Nogueira Calandrini de Azevedo, Sandra Maria do Valle Leone de Oliveira, Sheila Araújo Teles, Silvana Granado Nogueira da Gama, Sotero Serrate Mengue, Taynãna César Simões, Valdir Nascimento, Wildo Navegantes de Araújo

**Affiliations:** 1 Faculdade de Ciências Médicas da Santa Casa de São Paulo, Departamento de Saúde Coletiva, São Paulo, SP, Brasil; Universidade Federal do Espírito Santo, Vitória, ES, Brasil; Universidade Federal do Ceará, Departamento de Saúde Comunitária, Fortaleza, CE, Brasil; Faculdade Ciências Médicas Santa Casa de São Paulo, São Paulo, SP, Brasil; Secretaria de Estado da Saúde do Amapá, Macapá, AP, Brasil; Universidade Federal de Santa Catarina, SC, Brasil; Organização Pan-Americana da Saúde, Brasília, DF, Brasil; Instituto Evandro Chagas, Belém, PA, Brasil; Faculdade de Ciências Médicas Santa Casa de São Paulo, Departamento de Saúde Coletiva, São Paulo, SP, Brasil; Universidade Federal de Mato Grosso, Cuiabá, MT, Brasil; Universidade Federal do Paraná, Curitiba, PR, Brasil; Universidade Federal de Goiás, Goiânia, GO, Brasil; Universidade Federal do Piauí, Teresina, PI, Brasil; Universidade de Pernambuco, Faculdade de Ciências Médicas, Recife, PE, Brasil; Instituto de Saúde Coletiva, Universidade Federal da Bahia, Salvador, BA, Brasil; Secretaria de Estado da Saúde de Alagoas, Maceió, AL, Brasil; Universidade Federal do Acre, Rio Branco, AC, Brasil; Universidade Federal do Maranhão, Departamento de Saúde Pública, São Luís, MA, Brasil; Universidade Federal de Sergipe, Aracaju, SE, Brasil; Secretaria Municipal de Saúde, Boa Vista, RR, Brasil; Fundação Oswaldo Cruz, Mato Grosso do Sul, Campo Grande, MS, Brasil; Fundação Oswaldo Cruz, Escola Nacional de Saúde Pública Sergio Arouca, Rio de Janeiro, RJ, Brasil; Universidade Federal do Rio Grande do Sul, Porto Alegre, RS, Brasil; Fundação Oswaldo Cruz, Instituto de Pesquisa René Rachou, Belo Horizonte, MG, Brasil; Secretaria de Desenvolvimento Ambiental de Rondônia, Porto Velho, RO, Brasil; Universidade de Brasília, Brasília, DF, Brasil

**Keywords:** Cobertura Vacinais, Saúde da Criança, Inquéritos Epidemiológicos, Programa de Imunização

## Abstract

**Objetivo:**

Estimar e comparar as coberturas vacinais em crianças de até 2 anos de idade nascidas entre 2017 e 2018 nos municípios de São Paulo e Campinas, segundo o Inquérito de Cobertura Vacinal (ICV) e o Sistema de Informação do Programa Nacional de Imunizações (SI-PNI).

**Métodos:**

O ICV analisou registros de vacinação que foram comparados com as doses registradas no SI-PNI, divididas pela população-alvo.

**Resultados:**

Em São Paulo, segundo o ICV, em 2017 apenas a do Bacilo de Calmette-Guérin (BCG) (91,7%; IC_95_% 87,0;94,7) e a primeira dose da rotavírus humano (90,6%; IC_95_% 6,5;93,5) atingiram as metas; em 2018, apenas a BCG (93,4%; IC_95_% 89,5;95,8), a primeira dose da rotavírus humano (90,5% IC_95_% 85,3;94,0), a primeira dose da pneumocócica 10-valente (95,3%; IC_95_% 91,7;97,4), a primeira dose da meningocócica C (95,1%; IC_95_% 91,5;97,2) e a segunda dose da pneumocócica 10-valente (95,0%; IC_95_% 91,4;95,0). Em Campinas, apenas a BCG atingiu a meta em 2017 (93,0%; IC_95_% 88,8;95,7) e nenhuma em 2018. Segundo o SI-PNI, nenhuma vacina atingiu a meta em ambas as cidades.

**Conclusão:**

As coberturas vacinais ficaram aquém do esperado, e estimativas mais precisas são necessárias para o monitoramento adequado da situação vacinal infantil.

## Contribuições do estudo

**Principais resultados:**

As coberturas vacinais estiveram aquém das metas por ambas as fontes de dados (inquérito vacinal e sistema de informações), o que coloca em risco o controle das doenças imunopreveníveis nas duas cidades.

**Implicações para os serviços:**

Evidenciar a importância do monitoramento preciso para o controle das doenças imunopreveníveis e permitir estratégias para a manutenção de coberturas vacinais elevadas.

**Perspectivas:**

A necessidade de mais incentivos para a implantação e/ou o enfrentamento às barreiras do sistema informatizado é imprescindível para o monitoramento adequado da situação vacinal.

## INTRODUÇÃO

O conhecimento da cobertura vacinal da população infantil traz informações importantes como a avaliação da efetividade e eficiência do Programa Nacional de Imunizações (PNI). Esse programa foi instituído em 1973 e institucionalizado em 1975, com o objetivo de coordenar ações sistemáticas de vacinação em esfera nacional, intensificando as atividades de imunização no Brasil.^1,2^ A incorporação de novas vacinas no calendário de rotina aumentou a complexidade do PNI nas últimas duas décadas e trouxe novos desafios, como alcançar e manter altas coberturas vacinais.^3,4^

Desde a criação do PNI, avanços importantes ocorreram nos sistemas de informação desse programa, iniciando-se com os dados manuais até a informatização. A partir de 1980, os dados passaram a ser consolidados em planilhas de Excel com doses aplicadas do calendário nacional que, à época, incluía as vacinas contra difteria, tétano e coqueluche (DTP), sarampo, poliomielite oral e Bacilo de Calmette-Guérin (BCG). Eram dados limitados e totalizados por unidades federadas. Em 1994, iniciou-se o processo de informatização dos dados da vacinação no Brasil.^5^

Com o apoio do Departamento de Informática do Sistema Único de Saúde, foi desenvolvido o Sistema de Informação de Avaliação do Programa de Imunizações (Siapi), com registro de vacinação por doses aplicadas, agregadas por tipo de imunobiológico, grupo-alvo, tempo e lugar, mantido como único sistema de informação oficial de vacinação no país até 2012. Nesse ano, o Siapi foi aprimorado para plataforma Web (SiapiWeb) e ficou vigente para os registros de vacinação de rotina até dezembro de 2020, em alguns municípios, enquanto outros implantaram o registro nominal da vacinação.^5^ O SiapiWeb foi utilizado até a implantação completa do Sistema de Informação do Programa Nacional de Imunizações (SI-PNI). Este trouxe como inovação o registro individualizado (nominal) na perspectiva de corrigir limitações relacionadas ao sistema de dados agregados, para melhorar a precisão dos indicadores de vacinação. Até 2019, 85% das 38 mil salas de vacinas utilizavam o módulo Registro de Vacinados do SI-PNI.^5^

Reconhecendo a evolução e o processo de melhoria do sistema, cabe ressaltar que dados do SI-PNI permitem estimar coberturas vacinais pelo número de doses aplicadas, sem revelar a situação vacinal individual ou o percentual de crianças com esquema completo. Outras limitações, como erros na estimativa do tamanho da população-alvo nos anos intercensitários, podem subestimar ou superestimar o denominador, o que gera coberturas vacinais distorcidas.^6^

Dados de inquéritos domiciliares produzem estimativas mais precisas das coberturas e possibilitam a estimativa de crianças com o esquema completo. Tais dados também contribuem para a compreensão dos determinantes socioeconômicos associados à distribuição heterogênea da cobertura vacinal infantil e para a estimativa da proporção de susceptíveis e fatores relacionados à equidade de acesso ao PNI.^6^

Atualmente, o PNI enfrenta o desafio de retomar as metas de coberturas vacinais e, para tanto, é imprescindível que os sistemas de informação disponibilizem dados mais precisos. No Brasil, a queda da cobertura vacinal do calendário infantil teve início em 2012, acentuando-se a partir de 2016. Em 2020, a pandemia de covid-19 agravou esse cenário.^7^

O último Inquérito de Cobertura Vacinal (ICV 2020) nacional foi realizado nas capitais dos 26 estados, no Distrito Federal e em 12 municípios do interior e incluiu 37.801 crianças nascidas entre 2017 e 2018 e residentes em áreas urbanas. O ICV 2020 constatou que apenas 60,1% tinham recebido todas as vacinas que compõem o calendário vacinal até os 24 meses de idade.^8^

Estudos que compararam dados administrativos oficiais e oriundos de inquéritos de cobertura vacinal têm indicado diferenças importantes nas coberturas vacinais, que sinalizam possíveis falhas no registro de doses aplicadas e/ou na estimativa da população-alvo.^9^

O objetivo deste trabalho é estimar e comparar as coberturas vacinais em crianças de até 2 anos de idade nascidas entre 2017 e 2018 nos municípios de São Paulo e Campinas, segundo dados do ICV 2020 e do SI-PNI. 

## MÉTODOS

Neste estudo, duas fontes de dados foram utilizadas para calcular e comparar as coberturas vacinais do calendário do PNI até os 24 meses de idade: ICV 2020 e SI-PNI, para nascidos em 2017 e 2018, nos municípios de Campinas e São Paulo, localizados no estado de São Paulo. Ambas as cidades são de grande porte, com populações estimadas em 1.139.047 e 11.451.999 habitantes, de acordo com o Censo Demográfico 2022 do Instituto Brasileiro de Geografia e Estatística.^10^

### ICV 2020

Trata-se de um inquérito de base populacional baseada em uma coorte retrospectiva da vacinação da criança desde o seu nascimento até os 24 meses. A amostragem iniciou com a estratificação, em cada domínio de estimação, fundamentada em informações dos setores censitários do Censo Demográfico 2010, o que gerou 4 estratos socioeconômicos dos setores urbanos dos municípios (A, B, C e D – sendo A o de melhor e D o de pior condição socioeconômica). Em cada município, os pontos de coorte da estratificação foram diferentes, considerando a renda nominal do chefe de família, o percentual de chefes de família com renda superior a 20 salários mínimos e o percentual de chefes de família alfabetizados.

A lista de recém-nascidos, obtida via Ministério da Saúde, foi georreferenciada nos setores censitários de residência da mãe. O cálculo do tamanho da amostra baseou-se em cobertura vacinal de 70%, efeito de desenho 1,4 e nível de confiança 95%, resultando em 452 em cada estrato, o que totalizou um tamanho da amostra prevista 1.808 crianças em cada um dos municípios. Mais detalhes metodológicos estão descritos em publicação prévia.^8^

A coleta de dados foi feita por meio de visitas domiciliares, de junho de 2020 a maio de 2022, com entrevistadores treinados que registraram respostas em questionário estruturado e fotografias da caderneta de vacinação em um *tablet*. 

Foram calculadas as coberturas de todas as vacinas previstas no calendário oficial do PNI até os 24 meses de idade: BCG; hepatite B (hepB); pentavalente (penta: DTP + *hemophilus influenza* B + hepatite B) – primeira + segunda + terceira doses; poliomielite 1, 2 e 3 inativada (VIP: primeira + segunda + terceira doses); rotavírus humano (VRH: primeira + segunda doses); meningocócica C (MENC: primeira + segunda doses + reforço); pneumocócica 10-valente (pneumo 10: primeira + segunda doses + reforço); febre amarela (FA), sarampo, caxumba e rubéola (tríplice viral: primeira + segunda doses); hepatite A (hepA: primeira dose); varicela (VZ: dose única); poliomielite 1 e 3 (atenuada) (VOPb: reforço); e difteria, tétano e pertússis (DTP: reforço). 

Considerando que diferentes composições de vacinas são utilizadas para proteção das mesmas doenças, foi realizada a junção em uma variável para cada dose de vacina que faz parte do calendário do PNI.^11^ Tem-se como exemplo o cálculo da cobertura da vacina pneumocócica 10-valente (pneumo 10, aplicadas nos serviços públicos): foram somadas as doses da pneumo 3, a fim de contabilizar as vacinas aplicadas pelo setor público e por serviços privados.

As coberturas vacinais com doses aplicadas e os respectivos intervalos de confiança foram calculados no programa Stata®, versão 17, utilizando-se o módulo *survey analysis*. 

O ICV 2020 foi aprovado pelos comitês de ética em pesquisa com seres humanos do Instituto de Saúde Coletiva da Universidade Federal da Bahia, sob o Parecer nº 3.366.818, em 4 de junho de 2019, com Certificado de Apresentação de Apreciação Ética (CAAE) 4306919.5.0000.5030; e da Irmandade da Santa Casa de Misericórdia de São Paulo, sob o Parecer nº 4.380.019, em 4 de novembro de 2020, com CAAE 39412020.0.0000.5479.

### SI-PNI

O número de doses aplicadas de cada imunizante do calendário vacinal vigente foi obtido em outubro de 2023, no sítio eletrônico do SI-PNI,^12^ nos anos de 2017, 2018 e 2019, em cada faixa etária (<1 ano e 1 ano) para nascidos vivos em 2017 e 2018.

As coberturas vacinais foram calculadas dividindo o número de doses aplicadas de determinada vacina pela estimativa de sua população-alvo, multiplicando-se por 100. Dados do Sistema de Informações sobre Nascidos Vivos^13^ indicam 189.740 e 185.016 nascidos vivos em São Paulo e 21.651 e 21.339 em Campinas em 2017 e 2018.

No SI-PNI são registradas diferentes composições de vacina, por exemplo, tríplice viral e tetraviral. Para a totalização de doses aplicadas, foi necessária a soma das doses dos diferentes imunizantes utilizados para proteger das mesmas doenças, de acordo com orientações da Secretaria de Vigilância em Saúde do Ministério da Saúde.^14^

Por se tratar de dados de domínio público de acesso irrestrito, agregados anonimizados e apresentados de forma consolidada por município, ano e faixa etária, não foi necessária apreciação por parte de Comitê de Ética em Pesquisa.

### Indicadores de desempenho do PNI

Os indicadores coberturas vacinais e taxas de abandono foram utilizados para comparação dos dados do ICV e do SI-PNI.

### Coberturas vacinais

Para ambas as fontes de dados, foram consideradas adequadas as coberturas vacinais que atingiram valores iguais ou superiores a 90% para BCG e VRH e 95% para as demais vacinas.^5^

### Taxas de abandono

As taxas de abandono foram calculadas dividindo o número de crianças que iniciaram mas que não completaram o esquema de determinada vacina pelo número de primeiras doses aplicadas, multiplicado por 100. As taxas foram consideradas: altas, se maiores que 10%; médias, se entre 5% e 10%; e baixas, se menores que 5%.^14^

## RESULTADOS

A coleta de dados foi realizada no período de julho de 2021 a fevereiro de 2022, no município de São Paulo, e entre outubro de 2021 e abril de 2022, em Campinas. A amostra do ICV foi composta por 797 e 742 crianças nascidas no município de São Paulo (85,1% do previsto) e 912 e 862 nascidas em Campinas (98,1% do previsto) em 2017 e 2018.

### Município de São Paulo, crianças nascidas em 2017

Segundo o ICV, apenas as coberturas das vacinas da BCG (91,7%; IC_95_% 87,0;94,7%) e do rotavírus humano (90,6%; IC_95_% 86,5;93,5%) atingiram as metas do PNI. Em média, as coberturas estiveram 3,4 pontos percentuais (p.p.) abaixo das preconizadas, variando de +1,7 p.p. para BCG a -9,9 p.p. para MENC reforço (Figura 1). As taxas de abandono foram médias para os esquemas da vacina tríplice viral e VRH, e nenhum teve taxa de abandono alta (Figura 2).

**Figura 1:** Coberturas das vacinas previstas no calendário básico de vacinação, segundo dados do Inquérito de Cobertura Vacinal (ICV 2020) e do Sistema de Informações do Programa Nacional de Imunizações (SI-PNI), diferenças entre as fontes (ICV 2020 *versus* SI-PNI) e em relação às metas de coberturas (PNI), em crianças nascidas em 2017 no município de São Paulo

**Figura 2:** Taxas de abandono para os esquemas das vacinas tríplice viral, meningocócica C (MenC), pneumocócica 10-valente (Pneumo 10), pentavalente (Penta), rotavírus humano (VRH) e poliomielite 1, 2 e 3 inativada (VIP), segundo dados do Inquérito de Cobertura Vacinal (ICV 2020) e do Sistema de Informações do Programa Nacional de Imunizações (SI-PNI) em crianças nascidas em 2017 e 2018 nos municípios de São Paulo e Campinas

Segundo o SI-PNI, apenas a cobertura da vacina da BCG aproximou-se da meta (89,8%), não atingida por nenhuma das vacinas. A variação foi de -0,2 p.p. para BCG até -24,1 p.p. para a VOPb; e a média de -17,9 p.p. (Figura 1). As taxas de abandono foram altas para os esquemas penta e VIP e médias para os esquemas tríplice viral e VRH (Figura 2).

Para todas as vacinas, as coberturas constatadas no ICV foram maiores do que as calculadas com dados do SI-PNI. Considerando os ICs_95_%, apenas as coberturas das vacinas da BCG, da primeira dose da VRH e da pneumo 10 reforço não foram diferentes entre as duas fontes. A média da diferença das coberturas foi de +14,4 no ICV; a menor diferença na BCG (+1,9 p.p. no ICV) e a maior na FA (+82,0 p.p. no ICV) (Figura 1).

### Município de São Paulo, crianças nascidas em 2018

No ICV, apenas as coberturas da BCG, da primeira dose da VRH, da primeira dose da pneumo 10, da primeira dose da MENC e da segunda dose da pneumo 10 atingiram as metas do PNI: 93,4% (IC_95_% 89,5;95,8%), 90,5% (IC_95_% 85,3;94,0%), 95,3% (IC_95_% 91,7;97,4%), 95,1% (IC_95_% 91,5;97,2%) e 95,0% (IC_95_% 91,4;95,0%). Em média, as coberturas estiveram 2,8 p.p. abaixo das preconizadas. As taxas de abandono foram altas para o esquema da vacina tríplice viral e média para a pneumo 10 (Figura 2).

Segundo o SI-PNI, nenhuma vacina atingiu a meta, assim como o observado para os nascidos em 2017, com a mesma fonte de dados. Em média, as coberturas estiveram 15,9 p.p. abaixo das preconizadas (Figura 3). As taxas de abandono foram médias para o esquema da vacina tríplice viral e negativas para pneumo 10 e penta, mostrando mais registros da última do que da primeira dose do esquema, na mesma faixa etária (Figura 2).

**Figura 3:** Coberturas das vacinas previstas no calendário básico de vacinação, segundo dados do Inquérito de Cobertura Vacinal (ICV 2020) e do Sistema de Informações do Programa Nacional de Imunizações (SI-PNI), diferenças entre as fontes (ICV 2020 *versus* SI-PNI) e em relação às metas de coberturas (PNI), em crianças nascidas em 2018 no município de São Paulo

As coberturas no ICV foram maiores do que as do SI-PNI nos nascidos em 2018 (p < 0,05), para todas as vacinas. A média da diferença entre as duas fontes foi de +13,1 p.p. no ICV. A menor diferença foi no reforço da pneumo 10 (+5,3 p.p. no ICV) e a maior foi na FA (+37,2 p.p. no ICV) (Figura 3). 

O ICV mostrou ligeira melhora (+0,7 p.p.) quando comparada a média das coberturas vacinais entre nascidos em 2017 e 2018 no município de São Paulo. As maiores diferenças foram no reforço da vacina MENC (+4,4 p.p.) e na segunda dose da vacina tríplice viral (-3,9 p.p.); considerando os ICs_95_%, não houve diferença estatística entre as coberturas nos dois períodos. 

Segundo o SI-PNI, de 2017 a 2018, houve aumento +2,0 p.p. na média das coberturas, com grandes variações: -20,1 p.p. para DTP a +45,4 p.p. para FA (Figuras 1 e 3).

### Município de Campinas, crianças nascidas em 2017

No ICV, apenas a cobertura da vacina da BCG (93,0%; IC_95_% 88,8;95,7%) atingiu a meta. Em média, as coberturas estiveram 3,7 p.p. abaixo das metas, variando de +3,0 p.p. para BCG a -12,5 p.p. para o primeiro reforço da pneumo 10 (Figura 4). As taxas de abandono foram médias para o esquema da vacina tríplice viral e baixas para os demais esquemas (Figura 2).

**Figura 4:** Coberturas das vacinas previstas no calendário básico de vacinação, segundo dados do Inquérito de Cobertura Vacinal (ICV 2020) e do Sistema de Informações do Programa Nacional de Imunizações (SI-PNI), diferenças entre as fontes (ICV 2020 *versus* SI-PNI) e em relação às metas de coberturas (PNI), em crianças nascidas em 2017 em Campinas

No SI-PNI, nenhuma cobertura vacinal atingiu a meta, com diferença média de -30,2 p.p., variando de -8,0 p.p. para a HepB até -75,1 p.p. para a FA (Figura 3). As taxas de abandono foram altas para os esquemas da penta e VIP e médias para os esquemas da pneumo 10 e VRH (Figura 5).

**Figura 5:** Coberturas das vacinas previstas no calendário básico de vacinação, segundo dados do Inquérito de Cobertura Vacinal (ICV 2020) e do Sistema de Informações do Programa Nacional de Imunizações (SI-PNI), diferenças entre as fontes (ICV 2020 *versus* SI-PNI) e em relação às metas de coberturas (PNI), em crianças nascidas em 2018 em Campinas

A diferença entre as coberturas obtidas pelas duas fontes também foi grande, em média +26,5 p.p. no ICV. A menor diferença foi constatada na vacina HepB (+4,6 p.p. no ICV), e a maior na FA (+71,7 p.p. no ICV), seguida da varicela (+31,4 p.p. no ICV) (Figura 4). Considerando o IC_95_%, não houve diferença estatística apenas na cobertura da HepB entre as duas fontes.

### Município de Campinas, crianças nascidas em 2018

Segundo o ICV, as metas foram alcançadas para nenhuma das vacinas previstas até os 24 meses de idade. A menor diferença em relação às metas foi constatada para a BCG (-0,7 p.p.) e, as maiores diferenças, para a VZ (-10,2 p.p.) e a HepA (-10,3 p.p.) (Figura 4). As taxas de abandono foram baixas para todos os esquemas (Figura 2).

No SI-PNI, a diferença média das coberturas para as metas foi -27,6 p.p., menor na vacina HepB (-3,9 p.p.) e maiores na VOPb (-40,0 p.p.), na FA (-40,8 p.p.) e no reforço da DTP (-54,0 p.p.) (Figura 5). As taxas de abandono foram altas para os esquemas da vacina tríplice viral e penta e média para o da VRH (Figura 5).

Assim como o observado para os nascidos em 2017, as coberturas no ICV foram maiores do que no SI-PNI nos nascidos em 2018 para quase todas as vacinas, com exceção da vacina HepB (+4 p.p. no SI-PNI). Em média, as coberturas constatadas no ICV foram 22,1 p.p. maiores do que as do SI-PNI, com maiores diferenças na VOPb (+32,3 p.p. no ICV), na febre amarela (+33,6 p.p. no ICV) e na DTP (+46,7 p.p. no ICV) (Figura 4). 

Ao contrário do que ocorreu em São Paulo, a maior parte das coberturas foi menor nos nascidos em 2018 em Campinas, segundo os dados do ICV (Figuras 4 e 5). A diferença média foi de -1,8 p.p., e as maiores diferenças foram no reforço da pneumo 10 (+5,6 p.p.) e na VZ (-6,1 p.p.); considerando os ICs_95_%, não houve diferença estatística entre as coberturas das vacinas nos 2 anos. Segundo o SI-PNI, as coberturas nos nascidos em 2018 foram melhores (+2,6 p.p.), com as maiores diferenças na vacina FA (+34,3 p.p.) e na DTP (-13,3 p.p.). 

## DISCUSSÃO

No município de São Paulo, segundo o ICV, apenas 2 vacinas atingiram as metas em 2017 e 5 em 2018, entre elas a BCG. Em Campinas, apenas a BCG atingiu as metas em 2017 e nenhuma em 2018. Calculadas com dados do SI-PNI, nenhuma das vacinas atingiu a meta de cobertura em crianças de até 2 anos de idade nascidas em 2017 e 2018 nas duas cidades. 

Séries históricas das coberturas vacinais em crianças no Brasil, obtidas do SI-PNI, mostraram que, no período 2000-2015, todas as metas preconizadas pelo Ministério da Saúde foram alcançadas^15^ e, no período 2016-2018, isso ocorreu apenas com a BCG.^16^ Entre 2015 e 2017, os maiores recuos ocorreram nas vacinas da poliomielite (-21,2%), da hepatite A (-21,0%), da meningocócica C (-19,5%), do rotavírus humano (-20,2%), da penta (-19,8) e da hepatite B (-17,6%).^17^

É importante ressaltar que esse fenômeno não é localizado. Uma avaliação abrangente dos padrões globais de coberturas de 11 vacinas em 204 países destacou notável progresso entre 1980 e 2010, período em que mais crianças estiveram protegidas de doenças evitáveis por vacinas na história. Entre 2010 e 2019, período em que o desenvolvimento de novas vacinas foi amplamente bem-sucedido, o ganho nas coberturas foi mínimo. Em alguns locais, especialmente na América Latina e no Caribe, as coberturas vacinais diminuíram.^18^

Essas quedas podem ser atribuídas a vários fatores, impulsionados pelos movimentos antivacina^19^ com a disseminação de notícias falsas que contestam eficácia e atribuem reações adversas graves às vacinas.^20^ Há, também, a despreocupação em relação a doenças consideradas eliminadas ou erradicadas, pois muitos entendem que a vacina já não é mais necessária, visto que essas doenças não constituiriam ameaça à saúde das crianças.^21^

Deve-se considerar que o desabastecimento de alguns produtos, os desafios operacionais na execução adequada da vacinação, incluindo o registro preciso dos dados, e as dificuldades de acesso aos serviços de saúde são elementos que podem explicar as baixas coberturas vacinais. A compreensão desses fatores é fundamental para planejar novas abordagens para recuperar os altos índices de cobertura vacinal já alcançados.^17,22^

Comparando as duas fontes de dados, as coberturas de todas as vacinas previstas no calendário infantil até 24 meses de idade foram menores quando calculadas com dados do SI-PNI dos nascidos vivos em 2017 e 2018, nos dois municípios, com exceção apenas na cobertura da vacina contra hepatite B, em Campinas, em 2018. Esses resultados evidenciam dificuldades no sistema de registro eletrônico, que diminuem a precisão das coberturas vacinais infantis reportadas. 

A despeito de todos os avanços obtidos, grandes desafios são impostos nas distintas áreas do PNI, em função da complexidade de toda sua trajetória. A qualificação dos dados de imunização no país desenvolveu-se com a utilização do SI-PNI, mas importantes problemas ainda estão sendo identificados, o que pode afetar a qualidade dos dados registrados.^16^ O SI-PNI está em fase avançada de implantação no Brasil, contudo enfrenta desafios técnicos, burocráticos e operacionais, relacionados à capacitação dos profissionais, ao domínio da tecnologia, à manutenção do sistema e à garantia da confidencialidade das informações.^23^

Mesmo com incentivos para a implantação do sistema e suporte para capacitação disponibilizados pelo Ministério da Saúde, não houve adesão maciça na utilização do SI-PNI pelos municípios. Ainda há resistência em registrar os dados de forma individualizada, visto que o registro de doses agregadas era mais rápido. Os principais entraves relatados por estados e municípios incluem a dificuldade na transmissão dos dados para a base nacional e a lentidão no processamento pelo Departamento de Informática do Sistema Único de Saúde. Isso ocorre, em parte, devido à incompatibilidade do SI-PNI com os sistemas próprios utilizados por alguns municípios.^15^

A necessidade de acesso à internet em todas as salas de vacina, o registro adequado e oportuno de vacinação quando o sistema está sem acesso à internet e o impedimento de registros duplicados estão entre as principais dificuldades para a adesão dos municípios ao sistema. Ocorre que as salas de vacinas de um mesmo município não compartilham informações, o que possibilita que um indivíduo se cadastre e seja vacinado em diferentes unidades de saúde.^23^

Em virtude da maior complexidade para se efetuar os registros pelo sistema, verifica-se alimentação irregular, inadequada e inoportuna, ou seja, doses não registradas ou feitas com atraso. Há também constantes erros de digitação que comprometem a qualidade da informação. Essas inconsistências podem resultar em disparidades entre os dados locais e os números consolidados em nível nacional,^15^ além de imprecisões no registro. Isso impacta a construção dos indicadores de imunização, subestimando as coberturas.

Para a obtenção de estimativas mais precisas, destaca-se, também, a importância da qualificação dos profissionais envolvidos no tratamento dos dados no sistema de informações, o que contribui para o fortalecimento da capacidade operacional dos municípios. Cabe um esforço de capacitação permanente para análise dos dados inseridos e exportados no sistema de informações.^24^

Os resultados deste estudo devem ser interpretados considerando suas limitações. No ICV, a coleta de dados ocorreu durante a pandemia de covid-19, o que impactou as taxas de resposta e poderia afetar a precisão das estimativas. Destaca-se que o cálculo de pesos amostrais pós-estratificação contemplou diferenças nas respostas entre grupos populacionais e as minimizou. Por ter incluído crianças de áreas urbanas, impossibilita a extrapolação de dados, em especial para populações rurais, de municípios de pequeno porte e grupos minoritários. A principal vantagem do inquérito está na obtenção dos dados diretamente nas cadernetas de vacinação, com tomada fotográfica das cadernetas e posterior digitação dos dados por profissionais experientes no PNI. Os dados obtidos para cálculos das coberturas pelo SI-PNI são secundários, sujeitos a possíveis erros de digitação, incompletude ou pendências de inserção nos sistemas de informação de imunizações.

As coberturas vacinais em crianças de até 2 anos de idade estão aquém das metas estabelecidas pelo PNI. Embora inquéritos bem conduzidos possibilitem estimativas mais precisas e compreensão dos fatores que contribuem para essas reduções, sua realização demanda considerável investimento de tempo e recursos financeiros. É primordial que o sistema de informação seja aprimorado para fornecer estimativas mais precisas, possibilitando o monitoramento adequado da situação vacinal.
